# Observation of Quantum Zeno Blockade on Chip

**DOI:** 10.1038/s41598-017-13327-x

**Published:** 2017-11-01

**Authors:** Jia-Yang Chen, Yong Meng Sua, Zi-Tong Zhao, Mo Li, Yu-Ping Huang

**Affiliations:** 1 Department of Physics and Engineering Physics, Stevens Institute of Technology, Hoboken, New Jersey 07030 USA; 2 Center for Quantum Science and Engineering, Stevens Institute of Technology, Hoboken, New Jersey 07030 USA

## Abstract

Overlapping in an optical medium with nonlinear susceptibilities, lightwaves can interact, changing each other’s phase, wavelength, waveform shape, or other properties. Such nonlinear optical phenomena, discovered over a half-century ago, have led to a breadth of important applications. Applied to quantum-mechanical signals, however, these phenomena face fundamental challenges that arise from the multimodal nature of the interaction between the electromagnetic fields, such as phase noises and spontaneous Raman scattering. The quantum Zeno blockade allows strong interaction between lightwaves without physical overlap between them, thus offering a viable solution for the aforementioned challenges, as indicated in recent bulk-optics experiments. Here, we report on the observation of quantum Zeno blockade on chip, where a lightwave is modulated by another in a distinct “interaction-free” manner. For quantum applications, we also verify its operations on single-photon signals. Our results promise a scalable platform for overcoming several longstanding challenges in applied nonlinear and quantum optics, enabling manipulation and interaction of quantum signals without decoherence.

## Introduction

The quantum Zeno effect occurs when a coherently evolving quantum system is strongly coupled to external degrees of freedom, such as a detector, with the result that the evolution is suppressed or frozen^1–4^. Since its first observation in a trapped-ion system in 1990^5^, the Zeno effect has been exploited for “interaction-free” measurement^6–10^, by which a “bomb” can be probed by a photon without overlap between the two and, thus, without physical interaction. First appearing as a fascinating topic of fundamental interests, it has later been studied for exotic applications in optics, atomic physics, and quantum information, such as counterfactual quantum computing^11^. Recently, all-optical switching has been proposed and demonstrated based on the Zeno effect induced by two-photon absorption in atomic vapors^12,13^.

In the domain of nonlinear optics, we have analyzed^14–16^ and verified in two independent experiments^17,18^ an interesting Zeno phenomenon, called quantum Zeno blockade (QZB). QZB occurs when a photonic system interacts with external degrees of freedom through a nonlinear-optical channel, so that when the channel is fast (i.e., the nonlinear interaction is strong), occupation of a certain mode of the system can “block” (more precisely, suppress) additional photons from coupling into the system. Here, the nonlinear interaction functions as a continuous probe “monitoring” the system’s photon occupation, thus freezing its dynamics through the quantum Zeno effect. The channel can be dissipative, such as the two-photon absorption^12,19^, or coherent, such as sum or difference frequency generation^14,16^. In the case of a coherent channel, one can understand QZB by considering a simple three-level system with a signal and a pump wave^15^. The signal wave is initially in state |0〉. When there is no pump, it will coherently evolve into an orthogonal output state |1〉. However, by applying the pump wave which couples |1〉 to an ancillary state |2〉, the |0〉 ↔ |1〉 evolution will be disrupted, resulting in the signal being “frozen” in state |0〉. Thus the signal output will be switched between |0〉 and |1〉 depending on the presence or absence of the pump. Note that the signal and pump do not directly interact during this operation, as ideally the signal remains in |0〉 when the pump is on.

In the context of nonlinear optics, QZB can be understood by considering a system of a nonlinear Fabry-Prot cavity phase-matched for difference-frequency generation between a signal and a pump^15,17^. In the normal operation of the Fabry-Prot (without the pump), when a signal photon that is in the cavity resonance reaches the cavity, a tiny portion of its amplitude initially enters the cavity, and, upon successive round trips, constructively builds up and interferes with the incoming amplitude to allow the entire photon passing through cavity, i.e., with only a small overall reflection. With the pump on, however, the signal amplitude that enters the cavity is converted to its difference-frequency field, so the constructive interference is inhibited, thus preventing the signal photon from entering the cavity. From the Zeno perspective, the pump is constantly measuring if the photon is in the cavity, which causes it not to enter the cavity (nor interact with the pump), and will be reflected by the cavity instead.

One fascinating aspect of QZB, among others, is that it enables strong interaction between optical signals without them physically overlapping^14–16^. For example, using second-order nonlinear Fabry-Perot and whispering-gallery-mode (WGM) cavities, we have observed interaction-free all-optical switching, where a signal field is switched by a pump field only due to a *potential* for the nonlinear coupling between the two, but without such coupling actually happening in the asymptotic limit of very strong nonlinear effects^17,18^. Notably, unlike the photon blockade mediated by single atoms, here no excitation (such as excited atoms or excitons) is physically created in order for the blockade to take effect^20^. This distinct interaction-free and excitation-free features together eliminate the occurrence of phase noise, photon dissipation, and quantum state decoherence (e.g., via spontaneous emission)^15,16,21^, thus overcoming the fundamental challenges facing all-optical processing of quantum signals^22,23^. Moreover, we have found recently that implemented in a low-loss nonlinear microcavity, QZB allows deterministic interaction between single photons, which may give rise to scalable photonic quantum computing by avoiding post selection and use of auxiliary photons^21,24^.

**Figure 1:**
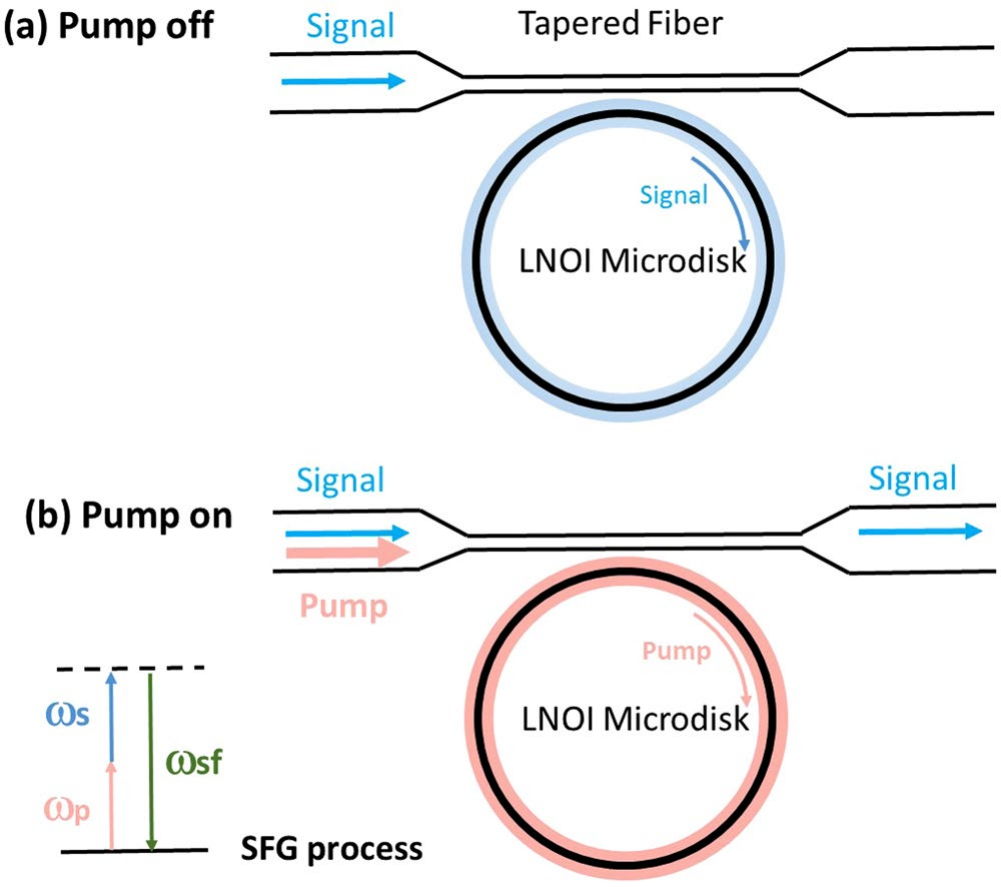
System schematic and operations in the pump off (**a**) and on (**b**) cases.

In light of these great promises, to date demonstrations of quantum Zeno effects have used atoms^5,12,13^, bulk-optical systems^17,18^, and superconducting qubits^25,26^. Here we demonstrate quantum Zeno blockade on a chip-integrated platform for scalable nonlinear and quantum optics applications^27,28^. The basic idea follows our previous studies in bulk optics^16,18^. As shown in Fig. 1, it consists of a WGM cavity nanofabricated on lithium niobate on insulator (LNOI) evanescently coupled to a fiber taper. The cavity is resonant with two nondegenerate lightwaves, namely the pump and signal, and phase matched for sum-frequency generation (SFG) between them. When the pump is off, the signal couples into the cavity and experiences strong loss in the cavity under the critical coupling condition. When the pump is applied, the resonance for the signal is altered due to the potential for SFG, so that it does not enter the cavity, therefore transmitting without cavity loss. The pump, on the hand, is not affected by the presence of the signal, as the latter does not enter the cavity in the asymptotic limit. This implements the QZB; see refs^15,16^ for detailed discussions, including a comparison of the system performance in different operational regimes. Using this system, we have observed strong Zeno blockade for interaction-free and excitation-free all-optical operations. As a validation of our system for quantum applications, we have also measured the noise level due to spontaneous photon scattering, and demonstrated all-optical modulation of single photons based on the Zeno effect. Our experiments mark a significant step toward scalable quantum information processing based on deterministic and noise-free logical operations for single photons.

The rest of this manuscript is structured as follows. In Section 1, we describe the LNOI nanocavity and characterize its nonlinear optical properties with second-harmonic generation. In Section 2, we present the Zeno blockade results for both continuous-wave and pulsed optical signals. In Section 3, we assess the background noise level of the current nanocavity and demonstrate interaction-free and excitation-free modulation of single photons. Lastly, we conclude in Section 4.

## LNOI nanocavity and second harmonic generation

**Figure 2:**
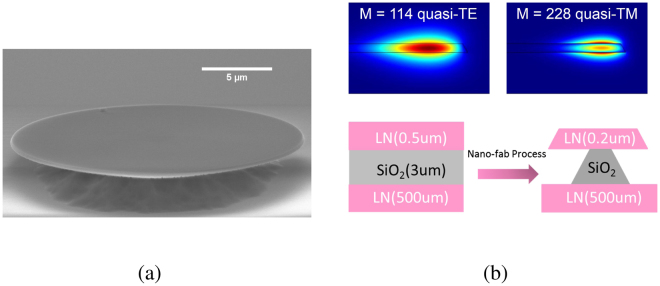
(**a**) Scanning electron microscopy (SEM) image of a LNOI microdisk with radius ~20 *μ*m. (**b**) Simulated profiles of phase matched cavity modes and fabrication schematic of the LNOI microdisk. M denotes the mode’s azimuthal order. The quasi-TE and quasi-TM modes are horizontally and vertically polarized relative to the microdisk plane, respectively, near 1550 nm and 775 nm.

The quantum Zeno blockade exploits the strong second-order nonlinear (*χ* ^(2)^) susceptibility of an air-suspended microdisk cavity nanofabricated on a z-cut LNOI, as shown in Fig. 2(a). The fabrication procedure is described in the Method section. For the present application, a major challenge exists with attainment of phase matching for the disparate wavelengths across the telecom C-band and near-IR band. To this end, we utilize both the strong birefringence of the lithium niobate material and the geometric dispersion of the strongly confined WGMs in the LNOI microdisk to offset the large chromatic dispersion of the interacting lightwaves over an octave. Specifically, we use quasi-transverse-electric (quasi-TE) modes for the telecom lightwaves and quasi-transverse-magnetic (quasi-TM) modes for the near-IR lightwave, and etch a 500-nm thick LNOI thin film down to only 200 nm using an inductively coupled plasma (ICP) system. With carefully designed parameters, we achieve natural phase matching for the second-harmonic generation between lightwaves near 1550 nm and 775 nm, each in the fundamental spatial mode along the cross-section. Figure 2(b) shows an example of such phase-matched modes based on a finite element method (FEM) analysis verified in our experiment. That the two modes correspond to alike optical fields with similar profiles, each strongly confined to sub-wavelength and singly peaked, gives rise to strong mode overlapping and hence offers superior nonlinear coupling between them^29^. The disadvantage with the current use of the birefringence, however, is that for the z-cut crystal orientation, the *χ* ^(2)^ processes are through the *d*_31_ and *d*_32_ tensor elements, which are 4 to 5 times lower than the largest element *d*_33_ of lithium niobate.

**Figure 3:**
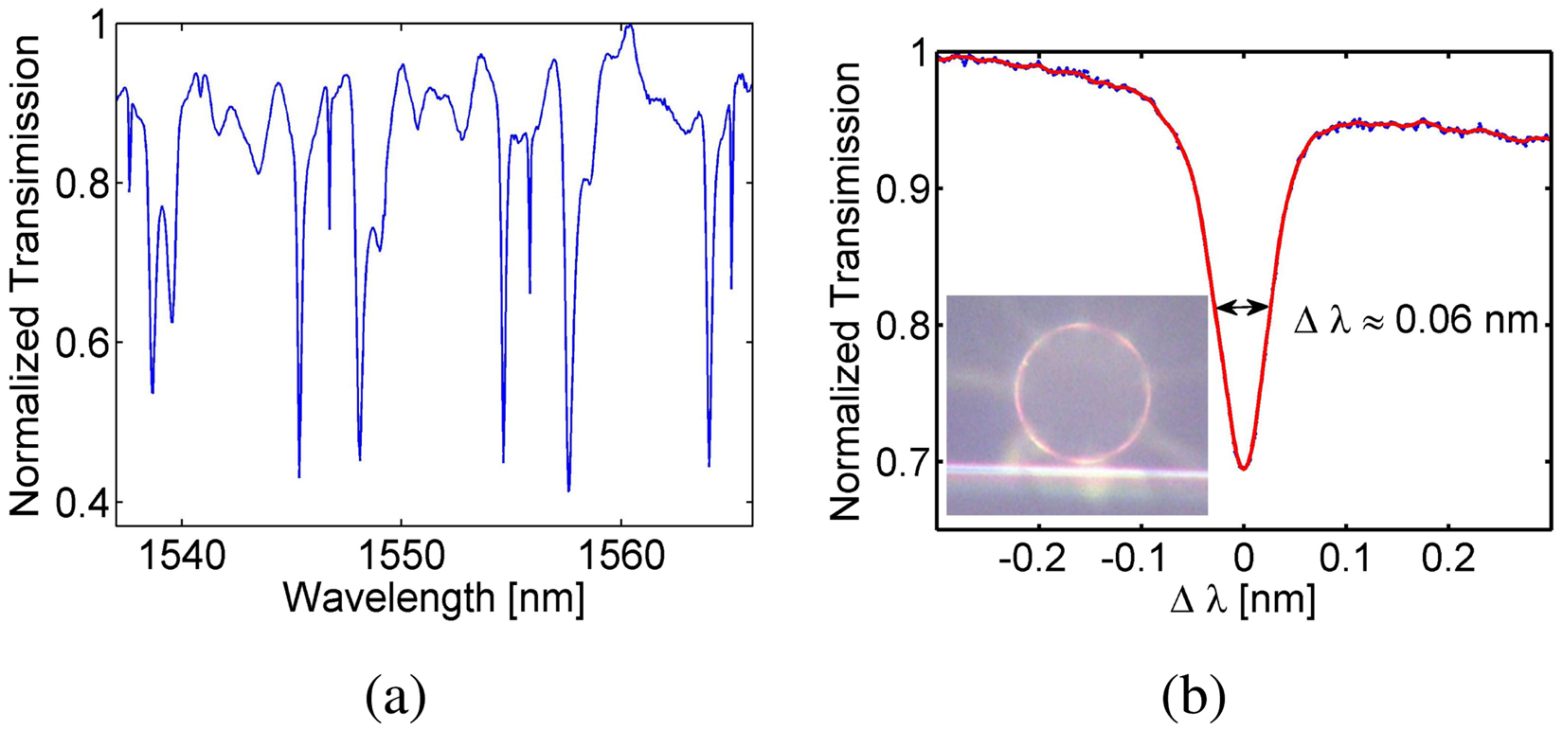
(**a**) The transmission spectrum of a LNOI microdisk. (**b**) The zoom-in spectrum of one mode which yields 2.6 × 10^4^ quality factor at around 1546 nm. The inset is the microscope image of the LNOI microdisk coupled with a tapered fiber.

For the on/off chip light coupling, we use a piece of tapered fiber as an evanescent coupler, as shown in Fig. 3(b). The fiber is tapered down to sub-micron diameter from a piece of standard single-mode fiber (SMF-28) using a home-built fiber-tapering system that incorporates flame brushing^30^. Its transmission loss can be as low as 0.05 dB upon tapering, but gradually increases to around 1 dB after extended exposure in ambient air. The coupling efficiency of the tapered fiber and the microdisk is sensitive to the relative position between them, so that we use a three-axis piezo positioning system to achieve a submicron tuning resolution.

To characterize the cavity resonances, we use an optical spectrum analyzer (OSA) to measure the transmission of a broadband lightwave generated through the amplified spontaneous emission (ASE) of an erbium-doped fiber amplifier (EDFA). A typical spectrum for a 20-*μ*m-radius microdisk is shown in Fig. 3, where the free spectral range (FSR) between the fundamental WGMs is measured to be 9.1 nm near 1546 nm. The smallest cavity linewidth is about 0.06 nm, which corresponds to a loaded cavity quality factor of 2.6 × 10^4^.

**Figure 4:**
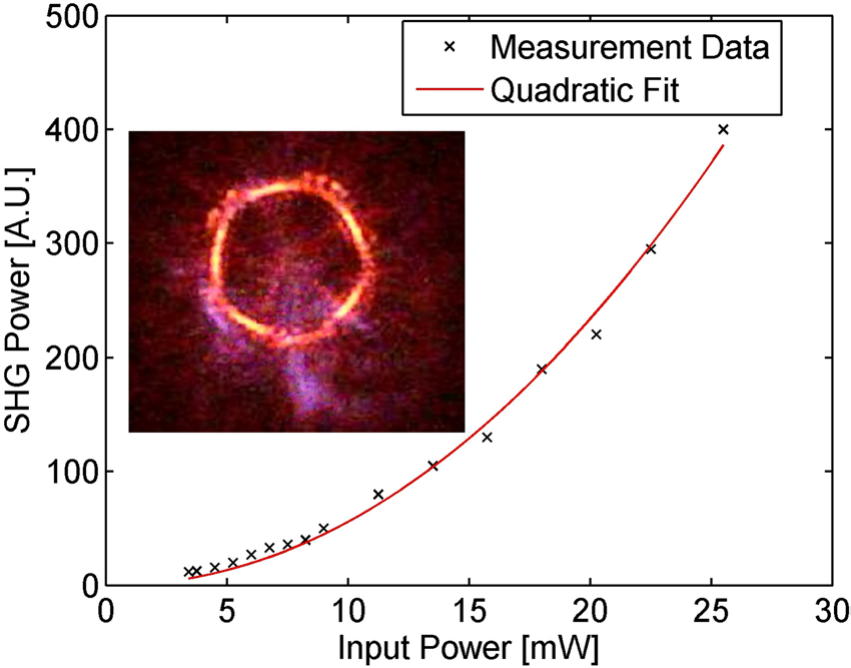
The power of the generated second-harmonic lightwave as a function of input pump power. Inset is the microscope image of the microdisk illuminated by the generated SH light.

To examine the phase matching, we measure the second-harmonic generation (SHG) in the microdisk by using a miliwatt continuous-wave (CW) pump at around 1546 nm light, which excites a fundamental quasi-TE mode. The generated second-harmonic light (around 773 nm) is coupled out of the microdisk through the same tapered fiber and measured by using OSA. The SHG power as a function of the input pump power is shown in Fig. 4, where a clear quadratic dependency is seen, thus verifying the frequency doubling process. Also shown in the figure’s inset is a micrograph of the generated SH light scattered off the microdisk.

## Experimental setup and Quantum Zeno Blockade

After the SHG, we switch to SFG between a CW pump and a CW signal wave to further optimize phase matching. It turns out that the strongest SFG occurs between two adjacent fundamental quasi-TE modes at 1536.790 nm and 1545.900 nm, respectively. For these CW lightwaves, however, the cavity resonances can thermally drift by as large as 0.1 nm, out of their respective resonance. In order to mitigate this thermal effect, we develop an optimization method for stabilizing the SFG, as detailed in Method section.

**Figure 5:**
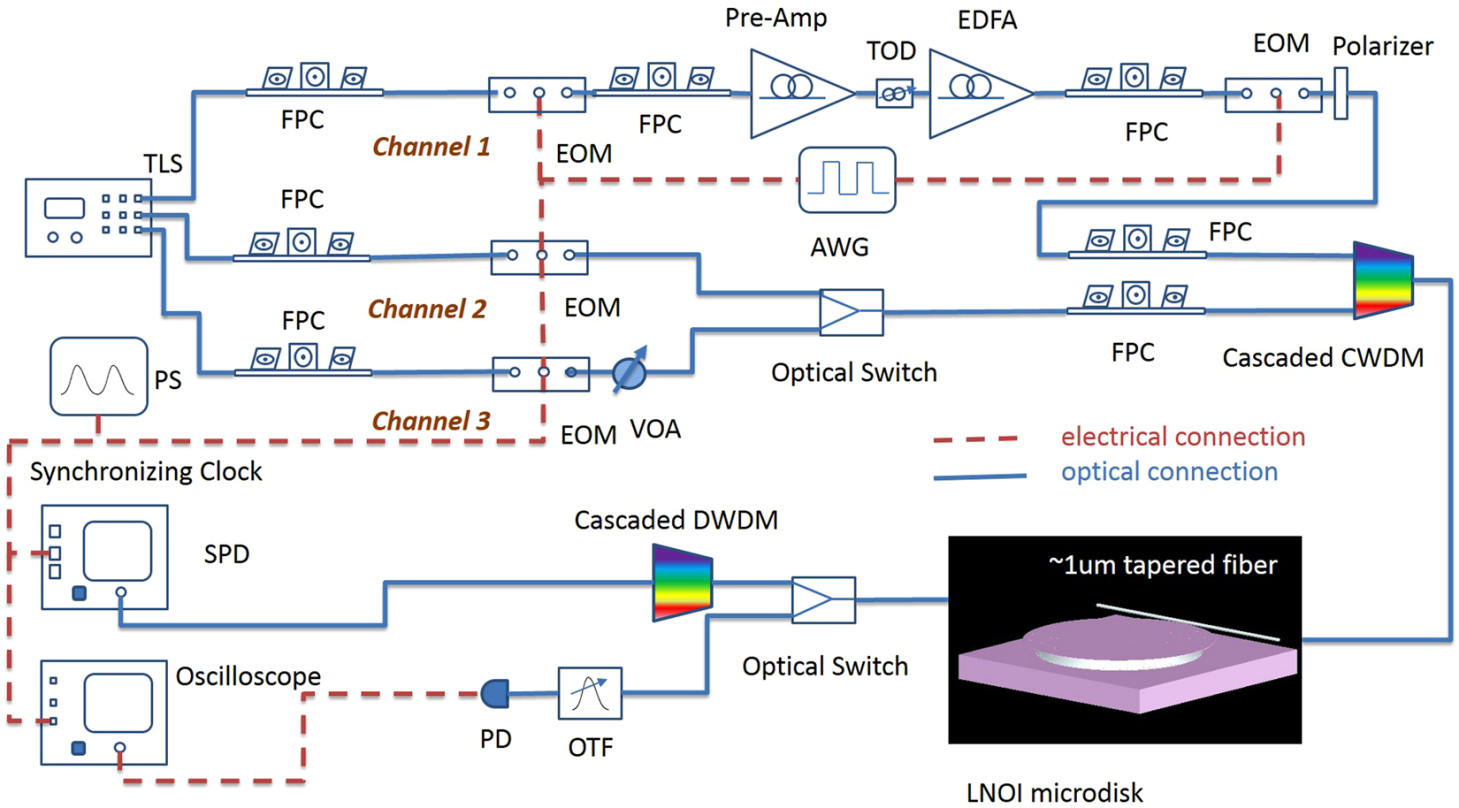
Experimental setup with three synchronized optical channels. Channel 1 creates a pump pulse train with 1 MHz repetition rate and 250 ps FWHM. Channel 2 produces a quasi-CW signal with 1-MHz repetition rate and 10-ns FWHM. Channel 3 generates signal pulses with a 50 MHz repetition rate and 200 ps FWHM. TLS, multichannel narrow linewidth (<100 KHz) tunable laser system, PS, electrical pulse generation system, EOM, electro-optic modulator, FPC, fiber polarization controller, VOA, variable optical attenuator, AWG, arbitrary waveform generator, CWDM, coarse wavelength-division multiplexer, DWDM, dense wavelength-division multiplexer, TOD, tunable optical delay, OTF, optical tunable filter, SPD, single photon detector.

Once efficient SFG is observed and thermally stabilized, we quickly swap in a quasi-CW signal and a pulsed pump at the same wavelengths, in order to obtain sufficient peak power for strong SFG while maintaining low average power to be around 250 *μ*W for the thermal stability. The experimental setup is shown in Fig. 5. The pump is a 1-MHz pulse train with 250-ps full width at half maximum (FWHM), created by modulating a narrow-band CW laser using two consecutive electro-optical modulators (EOM’s). To achieve the high pump peak power (~1 W), the output of the first EOM is amplified in a two-stage EDFA system, and pulse-picked by the second EOM to further lower the repetition rate to reduce the pump average power. The quasi-CW signal is synchronized and temporally aligned with the pump but having much wider pulse width (about 10 ns FWHM). The resulting pump and signal beams then each pass through a fiber polarization controller, before being combined into the microdisk through a pair of cascaded coarse wavelength-division multiplexers (CWDMs).

**Figure 6:**
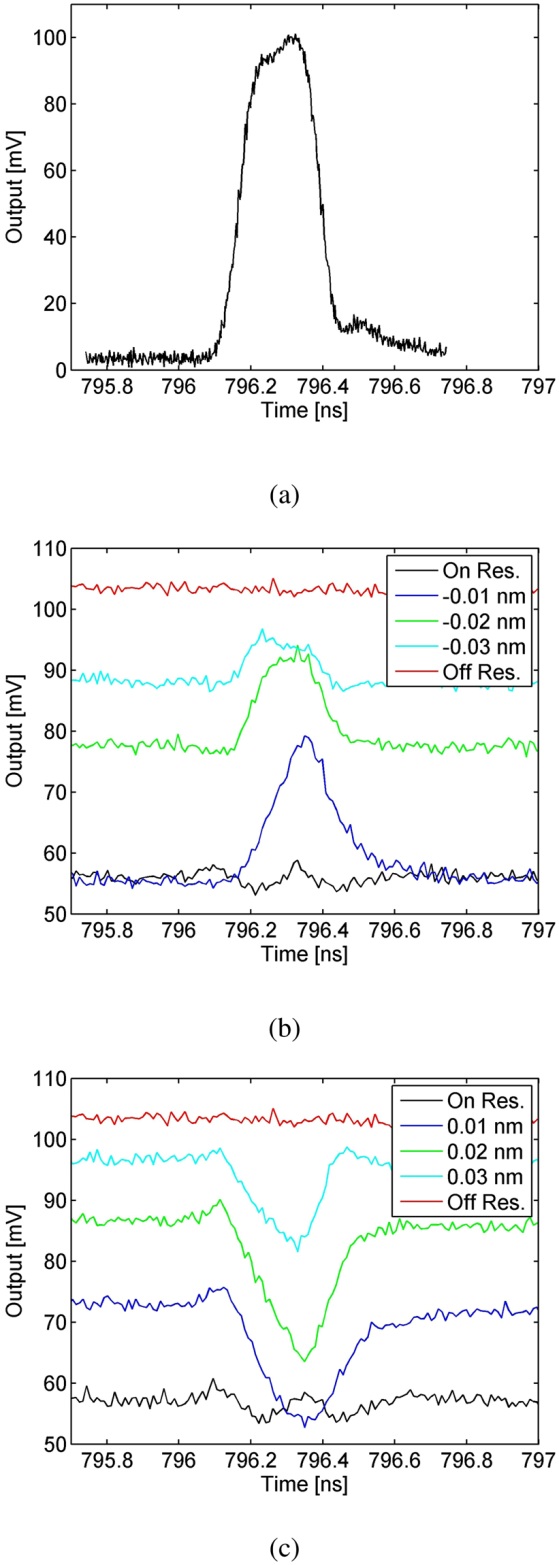
(**a**) Temporal profile of the pump pulse with about 250 ps FWHM. (**b**) QZB-induced modulation on a quasi-CW signal as it is blue detuned from its resonance center. The resonant wavelength is 1536.790 nm for the pump and 1545.900 nm for the signal. (**c**) The depletion of the signal under the same pumping condition but when it is red detuned. The 3-dB baseline of the transmitted signal even when it is right on resonance in Figure (**b**) and (**c**) is due to the under-coupling of the microdisk with the tapered fiber.

The average (peak) power coupled into the microdisk is approximately 170 *μ*W (680 mW) for the pump, and 4 *μ*w (400 *μ*W) for the signal. To account for the slight temperature change in the microdisk as we swap the CW with pulsed lightwaves, we fine tune the wavelengths of the pump and signal by less than 0.05 nm so that they remain hitting their respective resonance centers. Once adequate phase matching is achieved, the signal pulse overlapping a pump pulse will be deflected from the cavity due to QZB, as shown in Fig. 6(b). The highest modulation extinction—defined as the ratio between the QZB-induced reduction in the transmission loss to the loss when the signal is at the cavity-resonance center without QZB—is 51.0%, obtained when the signal is blue detuned from its resonance center by 0.01 nm. According to our simulation, this optimal wavelength for the signal is likely due to the imperfect phase mismatching, where the generated SF light is slightly off a resonance center; however, this warrants future studies. Further blue detuning the signal reduces the extinction ratio to 16.3% or less. In contrast, when the signal is red detuned, its depletion through the disk is enhanced by the pump, as shown in Fig. 6(c). These results show that the SFG driven by the pump effectively alters the signal’s cavity resonance while shifting it to a shorter wavelength; see^14,15^ for some detailed analyses. We note that in contrast to the thermally-induced resonance drift that affects all cavity lines, here the resonance altering depends critically on the phase matching condition for each cavity line. Thus a group of multiplexed signals can be selectively switched according to their resonant frequencies.

**Figure 7:**
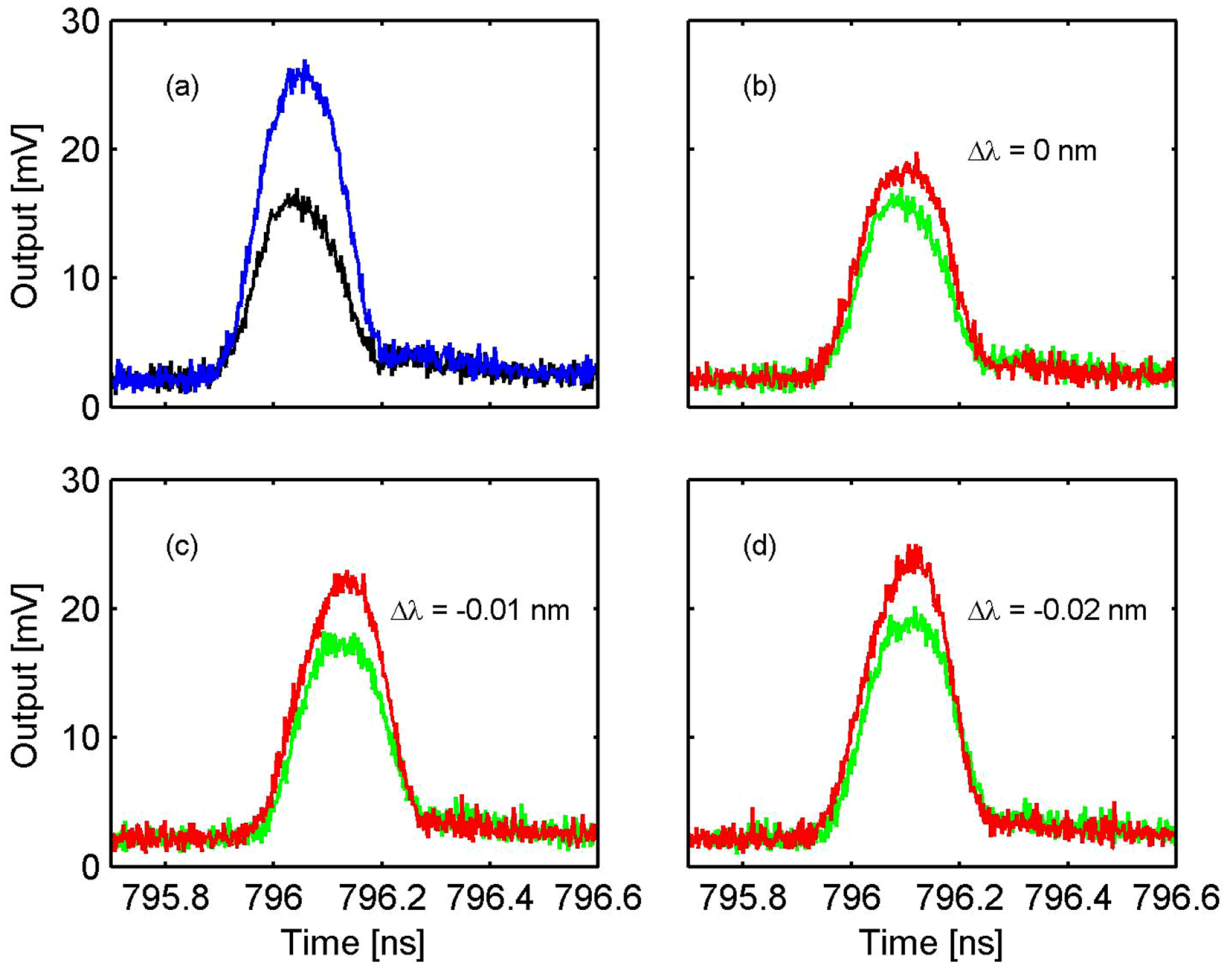
(**a**) The transmitted signal pulses in the on (black line) and off (blue line) resonance case, when they are not temporally aligned with the pump. (**b**–**d**) The signal transmission when they are blue detuned from the resonance center (1536.790 nm) by Δ*λ* = 0, 0.01, 0.02 nm, respectively. In each figure, the red line shows the result when the signal pulse is aligned with a pump pulse, while the green line shows that when the signal is completely displaced from the pump by 300 ps.

Next, we examine QZB for operations on pulsed signals. In order to modulate the entire signal pulses, we keep the pump width (250 ps FWHM) but narrower signal pulses to 200 ps FWHM. Furthermore, to easily distinguish QZB from any thermally-induced effect, we use different pulse repetition rates for the pump (1 MHz) and signal pulses (50 MHz), so that only one in every fifty signal pulses overlap with a pump pulse for QZB while the rest are unaffected. By keeping the same average power for the signal in the quasi-CW and pulsed cases, we are able to quickly swap them without disturbing the thermal equilibrium of the microdisk. The pump and signal are temporally aligned so that the peaks of the overlapping pulses are coincident within 10 ps. To contrast the signal transmission through the microdisk with and without QZB, we later use a tunable optical delay to temporally misplace the pulses. Figure 7 compares the transmission of the signal pulses as they are blue detuned from their resonance center. As seen, when there is no overlap between the signal and pump pulses, about 38.1% of the input signal power is lost in the microdisk when hitting the resonance center, because of the cavity’s under coupling. When the signal and pump are temporally aligned, the lost power is reduced as the signal is deflected from the cavity due to QZB. Specifically, at the resonance center, the signal loss with QZB becomes 29.3%, which corresponds to a 23.0% reduction. In contrast, when the signal is blue detuned by 0.01 nm, its transmittance loss becomes 14.4% with the pump and 32.8% without the pump, giving a 48.2% modulation extinction by the QZB. When the signal is further blue detuned to 0.02 nm, the extinction drops to 40.0%. This interesting variation of QZB effectiveness is because the SFG is not perfectly phase matched in the current microdisk, concordant with our numerical simulation. These results are consistent with our measurements in the quasi-CW case.

## Single-photon operations

We lastly assess the feasibility of our microdisk for quantum applications by quantifying its in-band photon noise level and demonstrating “interaction-free” modulation of single photons. Limited by our available filters (so as to attain the required >100 dB filtering extinction for the output signal photons), we select two non-adjacent fundamental quasi-TE WGM’s for the pump and signal at 1545.900 nm and 1564.750 nm, respectively. This wavelength selection unfortunately reduces the QZB efficiency by more than half as the SFG becomes less phase matched.

The experimental setup is shown in Fig. 5, where two CWDM filters are cascaded at the input to combine the pump and signal while rejecting their noises due to amplification spontaneous emission, and two dense-wavelength-division-multiplexers (DWDM’s) are cascaded at the output to pick the signal while rejecting the pump. Each DWDM filter is measured to have a 200-GHz FWHM bandwith and about 50 dB extinction. The resulting photons are counted using a commercial InGaAs single-photon detector (ID210, ID Quantique) with a 10.0% quantum efficiency, and the measurement is averaged over 2 million signal pulses.

In our setup, the noise photons can be created extrinsically in the fiber connecting the cascaded CWDM and DWDM filters through spontaneous Raman scattering, as well as intrinsically in the LNOI microdisk via spontaneous Raman scattering and spontaneous parametric down conversion (SPDC) of the second-harmonic lightwave created by the pump^31^. To quantify the intrinsic photon noise level, we first count the noise photons created in the fibers only, before the microdisk is coupled. Then, we bring the tapered fiber to the microdisk and count the photons again after verifying the fiber-microdisk coupling. The results are listed in Table 1, from which the intrinsic noise level is derived by comparing the photon counts with and without the microdisk. We note that the small difference in the two detector gating rates is due to the detector dead time (10 *μ*s) enforced after each photon detection, implemented to suppress the detector dark count.

**Table 1:** Noise Photon Counting with and without the microdisk.

Case	Gating Rate	Counting Rate	*P*_1_	*P*_2_
w/o microdisk	939.4 ± 1 KHz	6.65 ± 0.01 KHz	770 *μ*W	509 *μ*W
w/microdisk	935.6 ± 1 KHz	7.12 ± 0.01 KHz	776 *μ*W	332 *μ*W

Specifically, we first use the results without the microdisk to derive the number of noise photons created in a unit length of fiber per Watt of pump power, *R*_*f*_, through *R*_*f*_ *L*_1_ *P*_1_ *α*_1_ + *R*_*f*_ *L*_2_ *P*_2_ = *D*_1_/*ηG*_1_, where *L*_1_ = 3 m and *L*_2_ = 4 m are the fiber lengths before and after the microdisk, respectively, *P*_1_ and *P*_2_ are the input and output pump power, *G*_1_ and *D*_1_ are the detector gating rate and the photon counting rate. *α*_1_ is the linear loss in the tapered region, measured to be about 1.8 dB, and *η* is the total detection efficiency, which is 8.6% for the current setup, due to transmission loss and quantum efficiency of the single photon detector. Then, with the disk, we apply *R*_*f*_ *L*_1_ *P*_3_ *α*_2_ + *R*_*r*_ + *R*_*f*_ *L*_2_ *P*_4_ = *D*_2_/*ηG*_2_ to calculate *R*_*r*_, the rate of out-coupled noise photons created in the microdisk in the signal band. Here, *α*_2_ is the loss (3.7 dB) when coupled with the microdisk, *P*_3_ and *P*_4_ are the input and output power, respectively, and *G*_2_ and *D*_2_ are the gating and photon detection rates. This gives *R*_*r*_ = 9.3 × 10^−2^ per pump pulse, for the current pump pulse FWHM of 250 ps and peak power of 680 mW. As the DWDM filters combined give a spectral bandwidth of 200 GHz (FWHM), the number of detected time-frequency modes is about 13. Thus, by pushing to single-mode detection, the noise photon probability can be reduced to 7.2 × 10^−3^ per signal mode. By further detuning the signal and pump, the noise level can be suppressed to negligible^31^.

The relatively low intrinsic noise level of the LNOI microdisk—even for the present small detuning between the signal and pump—is due to its air suspended geometry^32^, isolated phonon modes^33^, and a relatively small Raman scattering cross-section^31,34^. Enhancing the sum-frequency generation using better mode overlapping or tailored pulse shapes will lower the pump power requirement and further suppress the background noise^35^. We expect that even for the pump and signal in the telecom C-band, upon improved nanofabrication techniques for a higher cavity Q, the level of noise photon can reach or exceed the dark count level of a typical InGaAs single-photon detector, which is about 10^−5^ per pulse.

We next demonstrate the QZB modulation of single-photon signals by using attenuated laser pulses, each containing 0.16 photons on average. Table 2 shows the measurement results as we blue detune the signal from the cavity resonance, after subtracting the total detection background, which is 7.12 KHz, attributing to the detector dark counts and the in-band noise photons created by the pump in the microdisk and fibers. Without QZB, when the signal is on (off) resonance, the photon detection rate and the gating rate are 59.20 KHz (62.88 KHz) and 465.0 KHz (395.0 KHz), respectively, yielding a photon probability 0.112 (0.159) (±1.0%). This amounts to a 29.6% transmission loss at the resonance center. With QZB, the loss is reduced with the highest modulation extinction is 20.6 ± 0.8%, obtained when the signal wavelength is blue detuned by 0.01 nm from resonance center, as consistent with our preceding results using bright signal pulses. By using optimally phase-matched wavelengths for the signal and pump, we expect to improve the extinction by more than two folds with the current microdisk.

**Table 2:** Summary of the single photon modulation results.

Signal detuning	w/o QZB	w/QZB	Extinction
0 nm	0.112	0.118	12.7%
0.01 nm	0.123	0.133	20.6%
0.02 nm	0.136	0.142	12.7%

## Conclusion

Using a nonlinear nanocavity, we have demonstrated quantum Zeno blockade on chip, where a light is modulated by another without them overlapping in the cavity in the asymptotic limit. This distinct “interaction-free” implementation overcomes several fundamental difficulties facing quantum nonlinear optics, including phase distortion, spontaneous Raman scattering, and pulse distortion, which would otherwise prevent achieving high performance for few-photon logical applications. Toward this end, we have shown that single photons can be modulated with low excessive noise. Our results pave a chip-integration approach to some unvisited areas in quantum nonlinear optics, where exotic operations are promised in a decoherence-free sub-space.

The next step is to improve the phase matching, including by applying centric periodic poling^36^, while replacing the current fiber taper with a waveguide coupler, based on which all-optical operations can be achieved at low error rate while cascaded on a single chip to realize complex functionalities. In addition, the current cavity quality factor is about 30,000. By increasing it to the vicinity of 10^8^, strong interaction between single photons can be realized, which, under the over-coupling condition, leads to “interaction-free” quantum logic gates with high fidelity^24^. Thus far, WGM resonators have been made in lithium noibate with cavity Q as high as 10^8^ in bulk optics^37^ and 10^6^ on chip^38^. With continuous advancement of fabrication technology, deterministic, scalable quantum information processing will be made possible via quantum Zeno blockade in the near future.

## Methods

### Microdisk fabrication procedure

The microdisk is fabricated on lithium niobate on insulator (LNOI, by NANOLN Inc.), which is a 500 nm lithium-niobate thin film bonded on a 3 *μ*m silicon dioxide layer above lithium niobate substrate. To attain good phase matching, we use FEM simulation tools to identify the optimum thickness of LNOI thin film to be 200 nm. Thus, 300 nm LN thin film is etched away using Oxford Plasmalab 100 Inductively Coupled Plasma (ICP) system. After piranha cleaning and dehydration, the etched sample with remaining 200 nm LN thin film is submerged into cationic organic surface active agent (SurPass 3000+) solution to improve surface adhesion with resist prior to spinning 1000 nm thickness of negative electron beam resist (ma-N 2410). Then the sample is pre-baked and patterned using EBL (Elionix ELS-G100, 100 keV). After development and post-bake, the sample is dry etched to obtain 200 nm thick microdisk by Argon milling process using the same ICP system. Buffered oxide etcher (BOE, 6:1) is used to undercut the microdisk to form the air-suspended structure. The chip is diced into smaller pieces for the measurement.

### Optimization method for stabilizing the SFG

To achieve locking-free yet efficient and stable SFG, we firstly use an optical spectrum analyzer (OSA) to measure the transmission of a broadband light from the amplified spectrum emission (ASE). Then the resonance centers of those fundamental quasi-TE polarized whispering-gallery-mode (usually the smallest linewidth) in each free spectrum range (FSR) are selected and marked. We switch in the CW light at each resonant wavelength and continuously fine tune its wavelength to compensate the thermally-induced shift in cavity resonance. Upon the cavity reaching its equilibrium status, we confirm its actual resonant wavelength via the brightness and the mode distribution of the generated SH light. Repeating the steps to check all those modes, we could identify and choose the best two modes for sum frequency generation. Lastly, we couple two CW lights at the wavelengths of the selected modes and apply the same method to track the moving resonance centers. Once efficient SFG is observed and thermally stabilized, we quickly switch to quasi-CW signal and pulsed pump while retaining their wavelengths.
